# Endovascular Repair of a Large Profunda Femoris Artery Pseudoaneurysm

**DOI:** 10.1155/2014/716752

**Published:** 2014-02-05

**Authors:** Ahsan Syed Khalid, Omar M. Ghanem, Seyed Mojtaba Gashti

**Affiliations:** ^1^Saba University School of Medicine, Devens, MA 01434, USA; ^2^Medstar Union Memorial Hospital, Baltimore, MD 21218, USA

## Abstract

Profunda femoris artery aneurysms and pseudoaneurysms are a rare cause of peripheral arterial aneurysms but their risk of rupture is quite high. We have presented a case of a left lower leg pseudoaneurysm. We have shown that endovascular repair with angioplasty and stenting is a suitable treatment method for such a pseudoaneurysm. Due to the limited data on this disease, we suggest multi-institute collaboration to identify and standardize management for the treatment.

## 1. Introduction

Profunda femoris artery aneurysms (PFAAs) are a rare cause of peripheral arterial aneurysms; however, the risk of rupture associated with such a finding is quite high. PFAAs are mostly asymptomatic and they usually present as an incidental finding. As for symptomatic patients, a swelling in the groin region is the most common presentation [1–3]. True aneurysms of the profunda femoris artery (PFA) are relatively rare (1–2.6%) and are idiopathic in nature without any suggestive cause. On the other hand, pseudoaneurysms of the PFA are more common and are generally secondary to, but are not limited to, orthopedic procedures, fractures, and penetrating or blunt trauma [2, 4]. We present a case of a PFA pseudoaneurysm in a patient with an orthopedic history.

## 2. Case Report

This is a case of a 38-year-old male who underwent intermedullary nailing of the left hip for avascular necrosis in September 2012. In March 2013, the patient presented with complaints of increasing edema of his left lower extremity associated with a palpable pulsatile mass in the anterolateral aspect of his thigh. At that point, the patient denied calf claudication on ambulation, nocturnal rest pain, or any other symptoms of the lower extremities. On exam, a palpable pulsatile mass in the anterolateral aspect of his thigh was noticed. He had a palpable thrill over it. Nevertheless, all lower extremity pulses were palpable.

**Figure 1:**
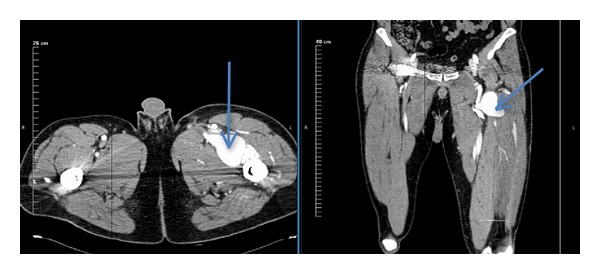
CT angiogram showing the left profunda femoris artery pseudoaneurysm.

**Figure 2:**
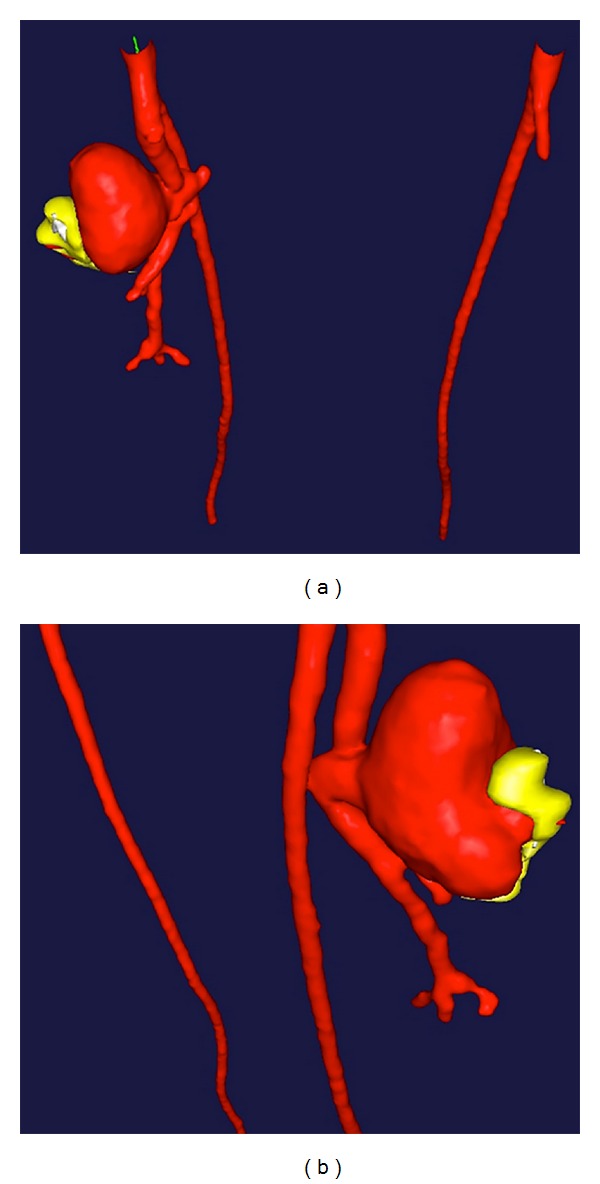
3D reconstruction of left profunda femoris artery pseudoaneurysm. (a) Posterior-superior view, (b) left anterolateral view.

A computed tomography (CT) angiogram (Figure 1) with 3-dimensional reconstructions (Figure 2) was obtained. The imaging revealed a left PFA pseudoaneurysm measuring 5 cm in AP diameter. The neck of the pseudoaneurysm was located 6 cm distal to the femoral bifurcation. In April 2013, the patient underwent exclusion of the left PFA pseudoaneurysm via covered stent placement.

## 3. Procedure

Right common femoral artery (CFA) access was obtained with a micropuncture needle. A crossover catheter was used to gain access to the left common iliac artery and a 7-French Ansel sheath was introduced over a stiff guidewire and parked in the left CFA. Selective angiography was performed again to precisely delineate the area of the pseudoaneurysm and its neck.

**Figure 3:**
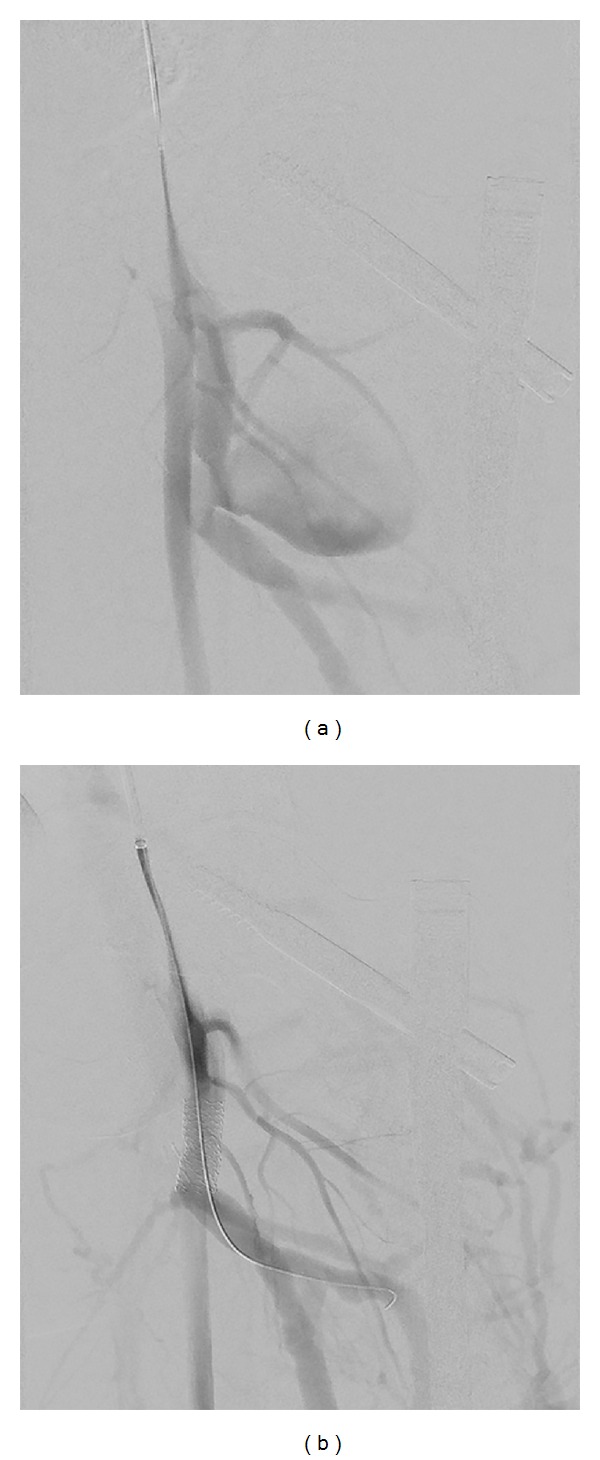
Angiogram of left lower extremity showing (a) the left profunda femoris pseudoaneurysm and (b) the poststent placement angiogram showing resolution of the pseudoaneurysm.

At this time, a 10 × 38 mm iCAST balloon expandable stent was deployed across the aneurysm and dilated using a 12 × 40 mm balloon. Angiography showed exclusion of the pseudoaneurysm; however, there appeared to be an endoleak from the proximal aspect. Therefore, a second stent was introduced in a similar fashion that overlapped the first one and extended more proximally. A repeat angiogram showed excellent results without any flow into the pseudoaneurysm (Figure 3).

## 4. Outcome

The patient tolerated the procedure well. One month followup showed significant decrease in the left lower extremity edema. There existed no associated pain or discomfort. Moreover, on his 6-month followup, the patient was clinically asymptomatic and had no bruit or thrills over the profunda femoris site.

## 5. Discussion

PFAA is an uncommon disease with limited data describing the course and optimal management. After reviewing several publications, solitary PFAAs account for roughly 0.5% of peripheral aneurysms [2, 5]. The risk of rupture in PFAAs is high and it is attributed to the large size of the aneurysm at the time of diagnosis. The increased size of the PFAA compared to other aneurysms such as a CFA and iliac vessel aneurysms is likely due to its deep location beneath the anterior muscles of the thigh. This makes the diagnosis difficult and thus leads to a delayed presentation [2, 6]. According to Tait et al., this increased size is the reason for the high risk of rupture of 30–45% of PFAAs relative to other peripheral aneurysms [7].

Most patients are asymptomatic and are diagnosed secondary to other diseases. Symptomatic patients most often present with a swelling of the upper thigh. Other symptoms of PFAAs include local compression of surrounding structures such as veins or nerves and thrombosis leading to ischemia and rupture. Moreover, PFAAs can serve as a distal source of emboli [1, 3, 8]. Our patient presented solely with a swelling of the left thigh with an associated palpable thrill. There were no other accompanying symptoms. The average size of PFAAs is difficult to document due to the rarity of the disease but a literature review by Posner et al. reported an average size of 7.4 ± 3 cm and an average age of 73.5 ± 10 years at time of presentation. In our 38-year-old male patient, the pseudoaneurysm measured 5 cm in AP diameter. Posner et al. reviewed 46 cases of PFAAs and found PFAAs to be more common in men (>92%) compared to women.

CT angiography and ultrasound are the appropriate imaging modalities used in the diagnosis of a PFAA [3, 6]. In addition, intraoperative intravenous ultrasound can be used to further characterize the pseudoaneurysm. In our case, we did not utilize intravenous ultrasound since the sizes of the pseudoaneurysm and the neck were characterized by preoperative CT angiography with 3D reconstruction. Previous studies have shown that 65–75% of PFAAs present with an accompanying aneurysm, namely, popliteal (47%), aortic (33%), and iliac (19%); thus, it would be beneficial to investigate for a PFAA in a patient who presents with an aneurysm elsewhere [1, 2]. Treatment options for PFAA should be geared towards removal of the risk of rupture as well as embolization, pain, and any compromised blood flow to the lower extremities. Upon diagnosis, the aneurysm should be repaired at the earliest convenience and reasonable recommendations suggest that repair should be considered immediately at a threshold of 2 cm or greater [1, 9]. However, most aneurysms have been and will be found at a size already greater than that since there is no indication to screen for PFAAs.

Standard treatment of the PFAA has been open surgery with either ligation of the PFA or reconstruction with a vein or a graft. The decision to undergo either one depends on the condition of the SFA. If the SFA is patent, then ligation of the PFA is permissible since there will be blood flow through the femoralpopliteal tract. However, it is suggested that it is best to repair the PFA rather than ligate, as this will maintain optimal flow to the lower extremity. If there is SFA disease or distal vascular disease, which can be associated with PFAA, it is a must to reconstruct the PFA to prevent ischemia to the lower limb, as the PFA is important for collateral circulation [1, 3, 9, 10].

Data on endovascular repair is limited with reports showing successful repair of a PFAA with endovascular coil embolization and others deploying covered stents to exclude the aneurysm [4, 6, 10, 11]. However, an embolization technique may be inappropriate in previous or current SFA occlusive disease [10, 11]. The literature suggests that PFA stenting is a good alternative [11]. Two published cases reported a ruptured PFAA that was successfully repaired with deployment of a stent [4, 10]. In our review of the literature, an endovascular approach was shown to be successful and is a less invasive alternative to open surgery [4, 6]. Although our patient had no history of SFA disease, we decided that endovascular repair with angioplasty and stent placement was the optimal route in management for our patient.

## 6. Conclusion

We reported a rare case of a pseudoaneurysm of the left PFA. On followup with our patient, he is doing well and there have been no complications with the procedure. We have shown that endovascular repair with angioplasty and stent placement is a reasonably safe and effective and a less invasive option for treatment compared to open surgery. Because of the limited data on endovascular repair of PFAAs, we suggest multi-institute collaboration to identify and standardize the management of this disease.
